# Pathogen Recognition and Activation of the Innate Immune Response in Zebrafish

**DOI:** 10.1155/2012/159807

**Published:** 2012-07-01

**Authors:** Michiel van der Vaart, Herman P. Spaink, Annemarie H. Meijer

**Affiliations:** Institute of Biology, Leiden University, Einsteinweg 55, 2333 CC Leiden, The Netherlands

## Abstract

The zebrafish has proven itself as an excellent model to study vertebrate innate immunity. It presents us with possibilities for *in vivo* imaging of host-pathogen interactions which are unparalleled in mammalian model systems. In addition, its suitability for genetic approaches is providing new insights on the mechanisms underlying the innate immune response. Here, we review the pattern recognition receptors that identify invading microbes, as well as the innate immune effector mechanisms that they activate in zebrafish embryos. We compare the current knowledge about these processes in mammalian models and zebrafish and discuss recent studies using zebrafish infection models that have advanced our general understanding of the innate immune system. Furthermore, we use transcriptome analysis of zebrafish infected with *E. tarda, S. typhimurium*, and *M. marinum* to visualize the gene expression profiles resulting from these infections. Our data illustrate that the two acute disease-causing pathogens, *E. tarda* and *S. typhimurium*, elicit a highly similar proinflammatory gene induction profile, while the chronic disease-causing pathogen, *M. marinum*, induces a weaker and delayed innate immune response.

## 1. Introduction

The use of adult zebrafish (*Danio rerio*) and their transparent offspring as hosts to model infectious diseases caused by human pathogens, or closely related animal pathogens, has recently provided novel insights into pathogenesis, which in many cases could not have been achieved using mammalian models [1–6]. The power of the zebrafish model lies in its suitability for genetic approaches, high-throughput screening, and live imaging studies. Fluorophore-marked transgenic lines are now available that allow unprecedented visualization of pathogen interactions with macrophages and neutrophils, the major phagocytic innate immune cell types of zebrafish larvae [7–11]. As early as one day after fertilization (dpf), zebrafish embryos display phagocytic activity towards microbial infections [12] and are able to mount an innate immune response with a transcriptional signature that resembles responses in mammalian or cell culture systems [13]. Adaptive immunity becomes active after approximately three weeks of development [14]. Therefore, innate immunity can be studied during the earlier zebrafish embryonic and larval stages in the absence of T- and B-cell responses. In this paper we focus on signaling pathways involved in pathogen recognition and activation of the innate immune response in zebrafish embryos and larvae. We compare the knowledge of the zebrafish innate immune system with that of human and mammalian models and discuss results from transcriptomic analyses that show clear specificity in responses to different bacterial pathogens, such as *Salmonella* and *Mycobacteria* species.

## 2. Pattern Recognition Receptors

**Figure 1:**
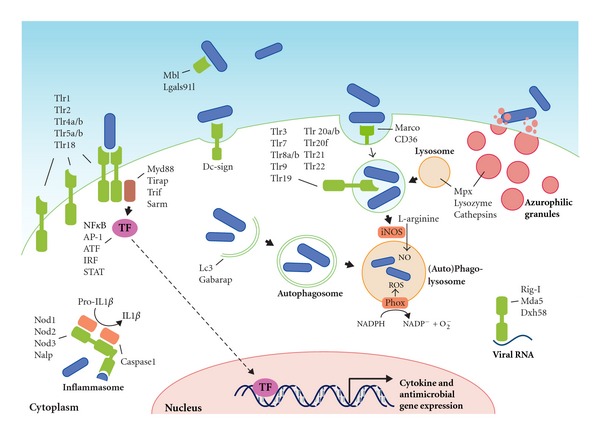
Pattern recognition receptors and effector mechanisms of the innate immune system. The localization of Tlrs on the cell surface or on endosomes is hypothetical and based on the known or proposed functions of their homologs in other fish or mammals. The ability of PRRs (depicted in green) to recognize PAMPs present on various types of microorganisms, like bacteria, viruses, and fungi, has been simplified here by depicting microorganisms as rod-like bacteria (in blue). PAMP recognition by PRRs leads to activation of transcription factors (TFs), which translocate to the nucleus and initiate transcription of cytokine genes, antimicrobial genes, and other immune-related genes. Defense mechanisms such as autophagy, ROS and NO production, and degranulation can be immediately activated upon microbial recognition, without de novo gene transcription.

The innate immune system is the host's first line of defense against infection; therefore, its main role is to recognize invading pathogens early and trigger an appropriate proinflammatory response [15]. The innate immune system utilizes a limited number of germline-encoded pattern recognition receptors (PRRs) to recognize evolutionary conserved structures on pathogens, named pathogen-associated molecular patterns (PAMPs) [15]. PRRs are also capable of indirectly sensing the presence of pathogens [16, 17]. This occurs when infection, inflammation, or other cellular stresses cause host factors to be present in aberrant locations, or to form abnormal molecular complexes, so called danger-associated molecular patterns (DAMPs) [17]. PRRs located on the cell surface are scouting the extracellular environment for the presence of microbes. PRRs located on endosomes identify microbes that have entered the phagolysosomal degradation pathway, and cytoplasmic PRRs recognize intracellular cytosolic pathogens or components of internalized microbes [18]. Upon PAMP recognition, PRRs signal the presence of infection and initiate proinflammatory and antimicrobial responses by activating several intracellular signaling pathways [19], ultimately leading to activation of gene expression and synthesis of a broad range of molecules. These include proinflammatory and chemotactic cytokines and antimicrobial peptides [20]. The different families of PRRs present in both humans and zebrafish and their downstream signaling pathways are summarized in Figure 1 and will be discussed below. 

### 2.1. Toll-Like Receptors

The most extensively studied class of PRRs are the Toll-like receptors (TLRs), a family of 10 proteins in human. TLRs are named after the *Drosophila *Toll protein, which functions in dorsoventral patterning and antifungal responses [23]. TLRs are integral glycoproteins which possess an extracellular or luminal, ligand-binding domain with leucine-rich repeat (LRR) motifs and a cytoplasmic signaling Toll/Interleukin-1 (IL-1) receptor homology (TIR) domain [20, 24]. In mammals, the main cell types expressing TLRs are antigen-presenting cells (APCs), including macrophages and dendritic cells, and B lymphocytes [18]. However, most cell types are capable of expressing TLRs, for instance, in response to a localized infection [25]. In mammals, TLR4 recognizes Gram-negative bacteria via the lipid A portion of lipopolysaccharide (LPS), while TLR2 recognizes Gram-positive bacteria via lipoteichoic acid (LTA), lipoproteins, and peptidoglycan, and TLR5 recognizes the motility apparatus protein flagellin, which can be present on both Gram types [18]. Other TLRs are specialized in recognizing nuclear acids in endosomal and phagosomal compartments. TLR3 can detect viral replication by binding to double-stranded RNA (dsRNA), TLR7 and TLR8 specifically recognize single-stranded RNA (ssRNA) of RNA viruses, and unmethylated CpG DNA present in the genomes of viruses and bacteria is detected by TLR9 [18]. Ligand binding by a TLR will induce it to form homomeric or heteromeric oligomers, which triggers intracellular signal transduction via their TIR domains [18]. The mammalian TLR signaling pathway uses five different TIR-domain-containing adaptor molecules: MYD88, MAL/TIRAP, TRIF/TICAM1, TRAM/TICAM2, and SARM [19, 24]. Among these, MYD88 is the most universal adaptor, since it is used for downstream signaling by all TLRs, with the exception of TLR3 [26]. Downstream signaling via central intermediate molecules such as TRAF6 will eventually lead to the activation of transcription factors, mostly members of the ATF, NF*κ*B, AP-1, IRF, and STAT families, regulating the expression of a battery of antimicrobial and proinflammatory genes [26]. 

Putative orthologs of mammalian TLRs have been identified in zebrafish, in addition to some fish-specific family members [27, 28]. A genome duplication during the evolution of teleost fish most likely explains why zebrafish have two counterparts for some of the mammalian TLRs (e.g., *tlr4ba/tlr4bb *for *TLR4* and *tlr5a/tlr5b* for *TLR5*), but it is still unknown whether this increase in the number of receptors is associated with diversification in PAMP recognition [4]. Only some of the zebrafish TLR ligands are currently known [29]. The specificity of TLR2, TLR3, and TLR5 is conserved between mammals and fish, recognizing lipopeptides, dsRNA, and flagellin, respectively [13, 30, 31]. Additionally, the fish-specific TLR22 has been shown to recognize dsRNA and PolyI:C [31]. However, zebrafish TLR4 cannot be stimulated by LPS, illustrating that not all ligand specificities are conserved between mammals and zebrafish [32, 33]. Signaling intermediates in the pathway downstream of mammalian TLRs have also been identified in zebrafish, including homologs of four of the adaptor proteins, Myd88, Mal/Tirap, Trif/Ticam1, and Sarm, and the central intermediate Traf6 [34]. Among these, Myd88 and Traf6 have been functionally studied by knockdown analysis in zebrafish embryos, showing their requirement for a proinflammatory innate immune response to microbial presence [13, 35–37]. Furthermore, triggering of the innate immune response in zebrafish embryos also leads to induction of members of the ATF, NF*κ*B, AP-1, IRF, and STAT families of transcription factors [13, 38]. 

### 2.2. NOD-Like Receptors

Pathogens that escape the surveillance of cell surface and endosomal PRRs may end up in the cytosol, where nucleotide-binding-oligomerization-domain- (NOD-) like receptors (NLRs) detect their presence by intracellular PAMPs and DAMPs [39]. The NLRs constitute a family of 23 proteins in humans. Their defining features are the presence of a centrally located NOD domain responsible for oligomerization, a C-terminal LRR capable of ligand-binding, and an N-terminal protein-protein interaction domain, such as the caspase recruitment domain (CARD), pyrin (PYD), or baculovirus inhibitor repeat (BIR) domain [40]. Two of the NLRs, NOD1 and NOD2, can sense bacterial presence by directly or indirectly detecting molecules produced during synthesis or breakdown of peptidoglycan [40]. NOD1 recognizes g-D-glutamyl-meso-diaminopimelic acid (iE-DAP), a dipeptide produced mostly by Gram-negative bacteria, whilst NOD2 can recognize both Gram types, since it is activated upon binding to muramyl dipeptide (MDP), a more common component of peptidoglycan [41, 42]. Interestingly, both NOD1 and NOD2 have recently been implicated in detection of parasites lacking peptidoglycan, indicating that these receptors can recognize a broader range of pathogens than was originally assumed [43, 44]. Upon ligand-binding, NOD1 and NOD2 recruit the serine/threonine kinase RIPK2 (also known as RIP2) via CARD-CARD interactions, eventually leading to the activation of NF*κ*B [45, 46]. In addition, NOD1/2 stimulation also induces MAP kinase signaling [47]. Synergistically, with TLR activation, NOD1/2 signaling cascades induce the expression of cytokines and chemokines, such as TNF, IL6, IL8, IL10, and IL12, as well as the production of antimicrobial peptides [46, 48, 49]. 

Other NLRs, such as IPAF, NALP1, and NALP3, mainly function to create a multiprotein complex known as the inflammasome, in which they associate with an adaptor called ASC (apoptosis-associated speck-like protein containing a CARD) and with procaspase 1 [50]. Oligomerization of the proteins in an inflammasome via CARD-CARD interactions ultimately leads to the cleavage of procaspase 1 into its active form, caspase 1, which is then available to catalyze the cleavage of accumulated pro-IL1*β* and pro-IL18 into their secreted forms, biological active IL1*β* and IL18 [40]. The NLR family member incorporated into these complexes determines which PAMPs and DAMPs are recognized by the inflammasome. A role for NALP3 has been established in the recognition of ATP [51], uric acid crystals [52], viral RNA [53], and bacterial DNA [54]. Both NALP1 and NALP3 share NOD2's ability to respond to MDP [55]. Furthermore, NALP1 can associate with NOD2 (Hsu 2008), showing a role for NOD2 in MDP-triggered IL1*β* activation, separate from its role as an inducer of proinflammatory gene expression.

Although the function of NLR family members in zebrafish is not widely studied, it is known that the canonical members of the mammalian NLR family, NOD1, NOD2, and NOD3 (or Nlrc3) are conserved. Additionally, a subfamily of NLRs resembling the mammalian NALPs and a unique teleost NLR subfamily are present [34, 56]. Confirmation of the antibacterial role of NOD1 and NOD2 in zebrafish was achieved by gene knockdown, resulting in higher bacterial burdens and decreased survival of embryos following *Salmonella enterica* infection [57]. Moreover, *nod1/2* depletion significantly decreased expression of dual oxidase (DUOX), required for production of reactive oxygen species (ROS) [57]. These findings illustrate that the family of Nod-like receptors and their downstream signaling pathways are important for antibacterial innate immunity, both in mammals and in zebrafish.

### 2.3. RIG-I-Like Receptors

Another family of cytosolic PRRs, the RIG-I-like receptors (RLRs), consists of three members: RIG-I (retinoic acid-inducible gene I), MDA5 (melanoma differentiation-associated factor 5), and LGP2 (laboratory of genetics and physiology 2). All three members are DExD/H box RNA helicases that can detect the presence of RNA from a broad range of viruses [58]. While expressed at low levels in most tissues, their expression is greatly increased upon viral infections or interferon (IFN) exposure [59, 60]. The RNA helicase domain of RLRs has the capacity to hydrolyze ATP and bind to RNA [61]. Furthermore, RIG-I and MDA5 contain a tandem of CARDs, which facilitate protein-protein interactions [60]. LGP2 lacks the two CARDs and is thought to function as a negative regulator of RIG-I and MDA5 signaling [62]. Following recognition of viral RNA, the CARDs of RIG-I and MDA5 become available for binding to a common mitochondrial signaling adaptor, IPS-1 or MAVS [63]. The subsequent signaling cascade culminates in the induction of transcription factors like interferon regulatory factor 3 (IRF3), IRF7, and NF*κ*B [64]. Activation of these transcription factors leads to the production of type I IFN, which binds to the IFN receptor to initiate expression of interferon-stimulated genes (ISGs) [65]. Amongst these ISGs are antiviral proteins, immune-proteasome components, all three RLRs, members of the TLR family, transcription factors like IRF7, and various proinflammatory cytokines and chemokines [65]. As such, the RLR-induced pathway works cooperatively with TLR signaling to prepare the cell for elimination of viral infections [58].

Zebrafish homologs of RIG-I, MDA5, and DXH58 were identified in a genome search [66]. However, *in silico* analysis of the predicted proteins revealed that the domain distribution differs between humans and zebrafish [66]. For instance, whilst human RIG-I contains two CARDs, one DExD/H domain and a Helicase C domain, zebrafish RIG-I consists of a single CARD and a DExD/H domain [66]. Whilst functional studies of the RLR pathway are scarce, it is clear that zebrafish and other teleosts possess a strong antiviral IFN system, which shares a common evolutionary origin with mammals [67, 68]. The mitochondrial RLR adaptor, IPS-1/MAVS, was recently cloned from salmon and zebrafish, and overexpression in fish cells led to a constitutive induction of ISGs [68]. Furthermore, MITA, another adaptor functioning downstream of IPS-1/MAVS and upstream of Tank-binding kinase 1 (TBK1), was cloned from crucian carp (*Carassius  auratus*) and shown to activate zebrafish IFN promoter gene constructs, dependent on IRF3 or IRF7 [69]. 

### 2.4. Scavenger Receptors

Scavenger receptors are a large family of transmembrane cell surface receptors, present on macrophages, dendritic cells, mast cells [70], and some endothelial and epithelial cell types [71]. Although originally defined for their role in uptake of low-density lipoproteins (LDL), they are now known to act as PRRs for a wide variety of PAMPs, like LPS, LTA, CpG DNA, yeast zymosan, and microbial surface proteins [72]. Commonly, PAMP binding to a scavenger receptor will induce the cell to directly phagocytose the pathogen [73]. Upregulation of scavenger receptor expression via TLR signaling can be a mechanism to increase phagocytic activity [74]. Moreover, scavenger receptors can also contribute to cytokine production as coreceptors for TLRs [75, 76]. Some of the C-type lectins, discussed below, also display scavenger receptor activity.

Based upon their multidomain structure, scavenger receptors are divided into eight subclasses (A-H) (Murphy 2005). Subclasses A and B are the most extensively studied, but members from other subclasses have also been shown to recognize bacterial PAMPs [72]. SR-A, the founding member of subclass A, functions as a phagocytic receptor for bacterial pathogens like *Staphylococcus aureus*, *Neisseria meningitides*, *Streptococcus pneumonia*, and *Escherichia coli* [77–79]. Macrophage receptor with collagenous structure (MARCO), another subclass A member with established PRR activity [80], functions as a phagocytic receptor for *S*. *pneumonia* [81] and *N*. *meningitidis* [82]. MARCO was shown to cooperate with TLR2 to trigger macrophage cytokine responses to the mycobacterial cell wall glycolipid trehalose dimycolate (TDM) and *Mycobacterium tuberculosis* [83]. CD36, the most prominent member of subclass B, is a sensor for LTA and diacylated lipopeptide (MALP-2) and also acts as a coreceptor for TLR2 [75]. CD36-mediated phagocytosis of *S*. *aureus* was shown to be required for initiation of TLR2/6 signaling [84]. SR-BI (or CLA-1), also in subclass B, can bind to LPS and was implicated in phagocytosis of both Gram-negative and Gram-positive bacteria [85]. As well as their antibacterial roles, CD36 and SR-BI are also known for increasing the pathogenesis of malaria and hepatitis C virus (HCV). CD36 can function as a receptor for erythrocytes that have been parasitized by *Plasmodium falciparum*, adhering these cells to the venular endothelium of various organs (Pluddemann 2007). Furthermore, SR-BI is used by *Plasmodium* sporozoites and HCV as an entry site into hepatocytes [72]. 

Many homologs of the mammalian scavenger receptor family can be identified in the zebrafish genome, but a systematic analysis is still awaited. A zebrafish homolog of human MARCO was identified as a specific marker for macrophages and dendritic cells from adult zebrafish [86], and this gene is also myeloid specific in zebrafish embryos [87]. Expression of the *cd36* gene was upregulated after exposing zebrafish to haemorrhagic septicemia rhabdovirus [88]. In contrast, *cd36* expression was downregulated by *Mycobacterium marinum* infection in adult zebrafish and larvae [22]. 

### 2.5. C-Type Lectins

The C-type lectin receptors (CLRs) are a large family of carbohydrate-binding proteins that are highly conserved amongst mammals [89]. The diversity of the CLR family is illustrated by the fact that up to 17 groups are present in vertebrates, with some consisting of soluble serum proteins, whilst others consist of transmembrane proteins. These are mainly expressed in myeloid cells (macrophages and dendritic cells) but also in natural killer cells [90, 91]. The best known CLR in serum is mannose-binding lectin (MBL), a member of the collectin class, which binds to a variety of sugar moieties present on viruses, bacteria, fungi, and protozoa and activates the complement system [92]. In terms of their function as PRRs, the transmembrane CLRs that are expressed on myeloid cells are the most interesting. Transmembrane CLRs can be divided into two groups: the mannose receptor family and the asialoglycoprotein receptor family [93]. CLRs recognize pathogens mainly via ligand binding to mannose, fucose, and glucan carbohydrate structures, which means that together they are capable of recognizing most classes of human pathogens [93]. Like scavenger receptors, CLRs can act as phagocytic receptors for nonopsonized bacteria, leading to their destruction in acidified phagolysosomes [73]. The best-studied member of the asialoglycoprotein receptor family is Dectin-1, which mediates phagocytosis of yeast and the yeast-derived protein zymosan [94]. Phagocytosis induced by CLRs like Dectin-1 is not only important for the lysosomal breakdown of pathogens, but also for antigen presentation [95, 96]. Besides their role in phagocytosis, CLRs can directly induce gene expression upon carbohydrate recognition. PAMP recognition by Dectin-1, Dectin-2, and macrophage-inducible C-type lectin (Mincle) ultimately leads to activation of NF*κ*B [97–99]. Where Dectin-1 associates with the kinase Syk to activate NF*κ*B [100], Dectin-2 and Mincle are dependent on Fc receptor *Υ*-chain as an adaptor molecule [98, 99]. Other CLRs, for example, DC-specific ICAM3-grabbing nonintegrin (DC-SIGN), induce specific gene expression profiles upon pathogen recognition by modulating TLR signalling [93]. When DC-SIGN recognizes mannose or fucose moieties on pathogens such as *Mycobacteria*, HIV-1, measles virus, and *Candida albicans*, it activates a Raf-1-dependent signaling pathway that modulates TLR-induced NF*κ*B activation, increasing the production of IL8 and anti-inflammatory IL10 production [101].

Only a few homologs of CLRs have been described in zebrafish. A homolog of the complement activating mannose-binding lectin (MBL) was associated with resistance against *Listonella anguillarum* [102]. Expression of another soluble lectin, *lgals91l,* is enriched in zebrafish embryonic myeloid cells and is dependent on the Spi1/Pu.1 transcription factor that plays a crucial role in myeloid cell development in vertebrates [87]. A membrane type collectin, CL-P1 (collectin placenta 1), was shown to be involved in vasculogenesis during zebrafish embryogenesis [103]. In humans, CL-P1 is mainly expressed on vascular endothelial cells and has been shown to act as a scavenger receptor mediating the phagocytosis of bacteria and yeast [104]. A putative homolog for DC-SIGN has recently been proposed and is upregulated in immune-related tissues following infection by *Aeromonas anguillarum* [105]. Finally, putative homologs for the mammalian C-type lectin NK cell receptors have been identified in zebrafish and are differentially expressed on cells from the myeloid and lymphoid lineages [106]. 

## 3. Effector Mechanisms of the Innate Immune Response in Zebrafish

While the adaptive immune system requires several days before reacting to invading microbes, the innate immune system consists mostly of defenses that are constitutively present and activated immediately upon infection (Figure 1). The general inflammatory response is a crucial innate defense mechanism. A state of inflammation is necessary for proper function of host defenses, since it focuses on circulating immune cells and antimicrobial components of the plasma at the site of infection. Below, we focus on the effector mechanisms involved in the cell-mediated part of the innate immune response. In addition, soluble serum proteins, including complement factors and other acute-phase proteins, make an important contribution to the innate defenses, and strong induction of their encoding genes has been observed in adult and embryonic zebrafish infection models [13, 36, 38, 107–109].

### 3.1. Secreted Peptides and Lipid Mediators of the Innate Immune Response

Cytokines, including interleukins, chemokines, and interferons, are small secreted proteins that steer the host's immune system into a cytotoxic, humoral, cell-mediated, or allergic response [110]. Since this paper focuses on innate immunity, we will mainly discuss the cytokines produced by or acting on phagocytic cells. A distinction can be made between cytokines that promote a state of inflammation and cytokines that are anti-inflammatory. The main proinflammatory cytokines produced by phagocytes are TNF*α*, IL1*α*, IL1*β*, IL6, and IL8 [110]. TNF-*α* is processed as a membrane-bound protein and, when required, the active soluble factor is cleaved off by the TNF-*α* converting enzyme (TACE) [111]. Similarly, IL1*α* and IL1*β* are synthesized as inactive precursors that are only secreted as active cytokines after inflammasome-mediated cleavage by caspase 1 [112]. The most potent anti-inflammatory cytokine in humans is IL10, which deactivates the proinflammatory cytokine production by macrophages and T cells [113]. The IL10/IL12 balance, maintained by cells of the innate immune system, determines whether adaptive immunity polarizes towards a Th1 (promoted by IL12) or Th2 response. A Th1 response, which activates the bactericidal activities of macrophages, is the most important for controlling intracellular pathogens. The single type II IFN, IFN*γ*, is also required for activating macrophage bactericidal functions, while type I IFNs (IFN*α* and IFN*β*) and type III IFN (IFN*λ*) function in mounting antiviral responses. Finally, eicosanoid lipid mediators also promote (e.g., prostaglandins and leukotrienes) or inhibit (e.g., lipoxins) inflammation, thus synergizing with or antagonizing cytokine functions.

Many of the cytokine subfamilies are conserved between zebrafish and mammals [34]. However, there has been extensive expansion and diversification of members of the chemokine gene family in zebrafish, and their specific functions are yet to be determined [114]. Several of the main cytokines, like IL1*β*, IL6, and IL10, have been cloned and characterized [115–117]. Furthermore, the zebrafish homolog of interleukin 10 receptor 1 (IL10R1) has recently been identified and seems to contain all the protein domains that are required for its function in anti-inflammatory signaling [118]. The proinflammatory chemokine IL8 (CXCL8) and it receptors, CXCR1 and CXCR2, are also conserved between mammals and zebrafish [119]. In addition, a second IL8/CXCL8 lineage has been identified in both zebrafish and common carp (*Cyprinus carpio*), and the chemotactic properties of carp IL8/CXCL8 molecules of both lineages were demonstrated by *in vitro* chemotaxis assays using carp leukocytes [120]. Both pro- and anti-inflammatory cytokines are upregulated upon infection of zebrafish embryos with pathogens such as *S*. *typhimurium* [13], *P*. *aeruginosa* [121], and *E*. *tarda* [122, 123].

The role of TNF during *Mycobacterium  marinum* infection of zebrafish embryos was studied by knockdown analysis of the TNF receptor (*tnfrsf1a*), which revealed that intracellular bacterial burdens, granuloma formation, and necrotic death of macrophages are increased in the absence of TNF signaling [124]. The importance of TNF signaling during *M*. *marinum* infection was further illustrated when the same model was used to show that a strict balance between pro-inflammatory TNF and anti-inflammatory lipoxins is vital for control of mycobacterial infections, with either too much or too little TNF expression leading to a more severe outcome of the disease [1]. Another study using the zebrafish model indicates that TNF-*α* is a potent activator of endothelial cells, leading to the production of chemokines, whilst it has little effect on the activation status of phagocytes [125]. This suggests that fish TNF-*α* mainly functions in the recruitment of leukocytes to the site of infection, rather than activating them. 

The three IFN groups present in humans are not conserved unambiguously in zebrafish and other fish species. The type II group of IFNs in zebrafish consists of IFN*γ*1 and IFN*γ*2 [126]. Expression levels of the corresponding genes did not change upon infection of zebrafish embryos with *E*. *coli* or *Y*. *ruckeri*, but was increased by *M*. *marinum* infection [126, 127]. Viral infection induced their expression in adult zebrafish but not in embryos [126]. IFN*γ*1 and IFN*γ*2 were shown to bind to different receptor complexes, and Janus kinase 2a (Jak2a), but not Jak2b, was shown to be required for intracellular transmission of the IFN*γ* signal. Two groups of antiviral IFNs, named IFN*ϕ*1 and IFN*ϕ*2, exist in zebrafish, and structural analysis showed that these are evolutionarily closer to type I than to type III human IFNs [34, 67, 128]. IFN*ϕ*1 and IFN*ϕ*2 signal via distinct receptor complexes [67, 129]. All zebrafish IFN*ϕ* genes induce the expression of genes that are predicted to be involved in antiviral activities [67].

### 3.2. Phagocytosis, Autophagy, and Lysosomal Destruction

Internalization of microorganisms is triggered when they are recognized by phagocytic receptors, mainly by scavenger receptors discussed above. This type of direct phagocytosis is termed nonopsonic phagocytosis, while opsonic phagocytosis relies on host-derived proteins that coat the surface of the microbe thereby enhancing phagocytosis efficiency. Opsonins include complement fragments, most notably C3b, which are recognized by complement receptors [130]. Mannose binding lectin, which can initiate C3b formation, and antibodies that bind to Fc receptors (IgG) or that activate complement (IgM) are also considered opsonins. Regardless of which receptor initiates the process, phagocytosis requires the activation of kinases and Rab GTPases that control alterations in the phospholipid membrane and remodeling of the actin cytoskeleton [131]. In macrophages, fusion of the resulting vesicle with early and late endosomes will decrease the pH of the immature phagosome and alter the proteins present on its membrane. Ultimately, maturing phagosomes turn into phagolysosomes when lysosomes fuse with them, mixing their contents [132]. Lysosomes are highly acidic endocytic vesicles (pH < 5.5), containing active proteases and lipases, and hydrolytic enzymes such as cathepsin D [133]. In addition, phagolysosomes also contain bactericidal peptides (defensins) and have the ability to generate toxic oxidative compounds that help microbial degradation [134]. Most of our knowledge about phagosome maturation comes from studies of phagocytosis in macrophages, and much less is known about phagosome maturation in neutrophils. While macrophage phagosomes fuse with endosomes and lysosomes, neutrophil phagosomes obtain their bactericidal properties by fusing with secretory vesicles and granules [135, 136]. In contrast to phagosome maturation in macrophages, neutrophil phagosomes do not acidify in order to become microbicidal [135, 136]. 

Many intracellular pathogens, like *M*. *tuberculosis*, *S*. *typhimurium*, and *Legionella pneumophila*, have evolved the ability to prevent phagosome maturation in macrophages and survive inside these vesicles [137]. To a certain extent, such pathogens can also withstand the hostile environment of the (phago)lysosome. Other pathogens like *Listeria monocytogenes*, *Francisella tularensis*, and many viruses can escape the phagosome and enter the cytosol [138]. *Mycobacterium marinum*, a pathogen studied extensively in zebrafish to model human tuberculosis, can survive inside phagosomes but also escape into the cytosol and spread to neighbouring cells by actin-based motility [139, 140]. Phagosomal escape has also been observed for the human pathogen *M*. *tuberculosis* and is dependent on a virulence factor, the ESX-/RD1 secretion system, shared by all pathogenic mycobacteria [141]. Together, these data indicate that host cells face numerous pathogens that have developed multiple strategies to avoid the pathway of phagolysosomal degradation. To counter such threats, cells may use autophagy to clear microbes and microbe-containing vesicles from the cytosol. Autophagy is well known as a metabolic process that recycles nutrients by degrading intracellular organelles and proteins. Only recently, it has been recognized that autophagy also plays an important role in the innate immune response against intracellular pathogens [142]. Autophagy is initiated when an autophagosomal isolation membrane is formed around its target, enclosing it entirely in a double-membrane vesicle. This process relies on class III phosphatidylinositol 3-kinase (PI3-kinase) and autophagy-related genes (Atgs), such as Atg6 (or Beclin-1) [143]. The hallmark of autophagosomes is the presence of Atg8 (or LC3) in their membranes, which is essential for membrane elongation [144]. Similar to a maturing phagosome, the autophagosome also fuses with lysosomes to achieve its degradative properties [145]. In addition, autolysosomes acquire unique antimicrobial properties due to the function of autophagic adaptor protein p62, which delivers cytosolic components to autolysosomes where they are processed into potent antimicrobial peptides [146]. As reviewed elsewhere [147], pathogen-targeted autophagy can be induced by several TLRs and NLRs, TNF-*α*, NF*κ*B, and many other immune-related signalling molecules. 

**Figure 2:**
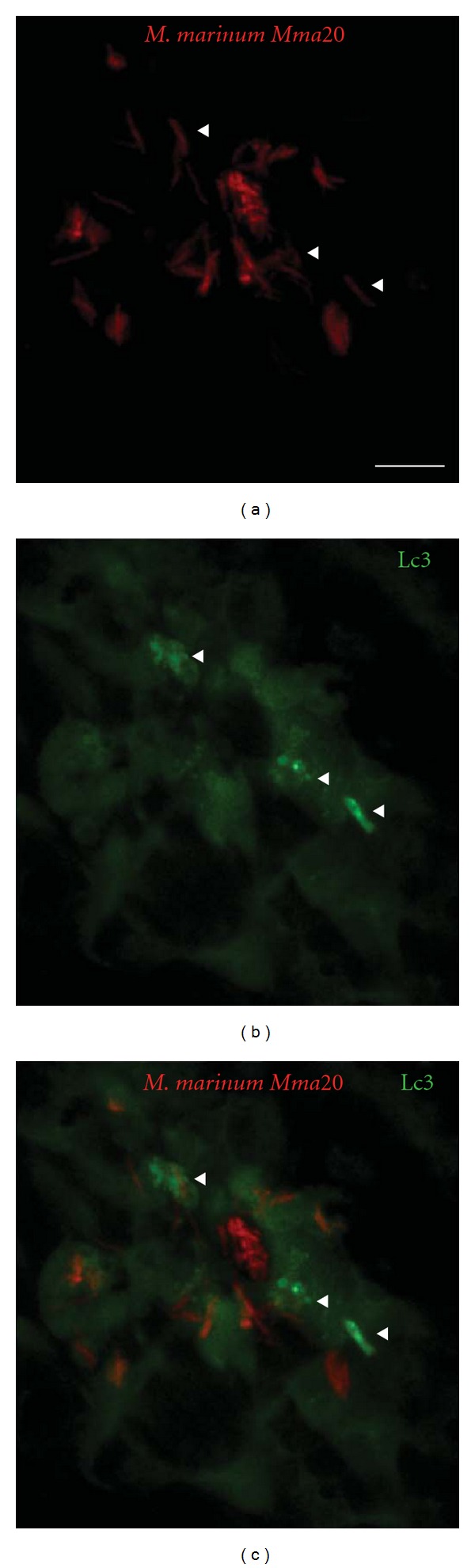
*In situ* detection of autophagy by Lc3 accumulation. CMV::LC3-GFP transgenic [15] zebrafish embryos (28 hpf) were injected into the caudal vein with 200 colony-forming units (CFU) of *M*. *marinum Mma20 *expressing a pMST3::mCherry vector. Confocal images were taken of a tail region of the developing larva at 3 days after infection (3 dpi), a point at which the *M*. *marinum* infection (a) has been established. Low levels of Lc3-GFP signal (b) can be observed throughout the cells, whilst brighter regions (indicated by arrowheads) are only observed upon Lc3 accumulation and formation of autophagic membranes associated with bacteria (c). Scale bar: 10 *μ*m.

The transparency of zebrafish embryos and availability of fluorescent macrophage and neutrophil reporter lines allow for study of the process of phagocytosis in great detail [7, 148–150]. It was recently shown that zebrafish embryonic macrophages efficiently engulf *E*. *coli* bacteria from blood- and fluid-filled cavities, while neutrophils are hardly capable of phagocytosing bacteria present in fluids [150]. However, neutrophils did prove to be highly phagocytic when moving over bacteria present on tissue surfaces. This shows that the type of immune cell that clears an infection not only depends on the PAMPs present on the invading microbe, but also on the characteristics of the infection site. An *in vivo* phagocytosis assay was used to show that functions of Wasp1, Wasp2, Abi2, and cofilin regulator 14-3-3*ζ* (Ywab) in bacterial phagocytosis are conserved in zebrafish [151]. The recent generation of a transgenic zebrafish line with GFP-tagged LC3 has enabled *in vivo *visualization of the interactions between microbes and this core component of the autophagy machinery [152]. The importance of autophagy in the innate immune response of zebrafish remains to be studied, but we have shown that LC3-labeled structures accumulate around *M*. *marinum* infection sites in zebrafish embryos (Figure 2). Furthermore, autophagy-related genes were induced in adult zebrafish infected with *Citrobacter freundii* and zebrafish embryos infected with *S*. *typhimurium* [37, 153]. 

### 3.3. Oxidative Defenses in Leukocytes

In several systems, it has been shown that neutrophils are the first immune cells to arrive at the site of infection or wounding. They facilitate their migration by exocytosing granules that contain metalloproteinases and other enzymes that degrade the extracellular matrix [154]. Upon recognition of pathogens, neutrophils release their antimicrobial granules, called azurophils, into phagosomes or the extracellular environment [155, 156]. Azurophils are packed with acidic hydrolases and antimicrobial proteins, such as lysozyme, cathepsins, and myeloperoxidase (MPO) [157]. The primary function of MPO is to react with hydrogen peroxide(H_2_O_2_),which subsequently oxidates chloride, tyrosine, and nitrite to form hypochloric acid (HOCl), tyrosine radicals, and reactive nitrogen intermediates [158]. These highly reactive chemicals attack the surface membranes of microbes. Additionally, microbes can be bound by neutrophil extracellular traps (NETs), which are fibrous networks of granule proteins and chromatin released by neutrophils [159].

While MPO is mostly produced in neutrophils, all professional phagocytes produce high levels of reactive oxygen species (ROS), including superoxide, H_2_O_2_, and hydroxyl radicals, produced by the enzymes NADPH oxidase (NOX) and dual oxidase (DUOX) [160]. The NOX of phagocytes (Phox) is only activated upon exposure to microorganisms or other pro-inflammatory stimuli [161]. When active, Phox is located in the phagosomal membrane and catalyzes the respiratory burst, which consists of the large-scale production of ROS that helps degrade phagocytosed microbes by nonspecifically oxidizing protein, DNA, lipid, and carbohydrate [162]. H_2_O_2_ produced during the respiratory burst can also function as a substrate for MPO activity. The oxidative enzyme DUOX may even combine the two functions, by generating H_2_O_2_ as a substrate for its own peroxidase domain [160].

Nitric oxide (NO) is produced from the amino acid L-arginine by nitric oxide synthase (NOS) enzymes and functions as a signaling molecule in numerous biological processes as well as having antimicrobial activity [163]. There are two constitutively expressed NOS enzymes, neuronal NOS (nNOS or NOS1) and endothelial NOS (eNOS or NOS3), and one inducible NOS (iNOS or NOS2) that is important in innate immunity. Regulation of NOS2 plays an important role in the inflammatory response, and many cells of the immune system are capable of producing NO [164, 165]. NO has cytostatic and cytotoxic antimicrobial effects when high amounts are excreted by immune cells into mammalian tissues, most likely via reactive nitrogen species (RNS) which are generated when NO interacts with O_2_ [166]. These RNS subsequently lead to lipid peroxidation, DNA damage, oxidation of thiols, and nitration of tyrosine residues [167]. It has recently been shown that Nos2a, the zebrafish homolog of NOS2, is also required for the expansion of hematopoietic stem cells and progenitor cells during infection, leading to increased numbers of the required immune cells [168]. This discovery further adds to the importance of NOS2 in the inflammatory response. 

The oxidative defense mechanisms need to be tightly controlled, since high levels of reactive chemicals like ROS and RNS cause tissue damage at sites of infection. Therefore, the resolution phase of inflammation is critical in order to restore the tissue to its normal state and prevent chronic inflammation. The molecules produced during oxidative defenses are often self-limiting and help initiate resolution of inflammation by inducing neutrophil apoptosis [160, 169]. Furthermore, iNOS-induced NO production can be countered by activation of arginase (ARG), which depletes the substrate for iNOS by converting L-arginine to the harmless compounds urea and L-ornithine, thus creating conditions more favorable for wound healing [163, 170]. 

**Figure 3:**
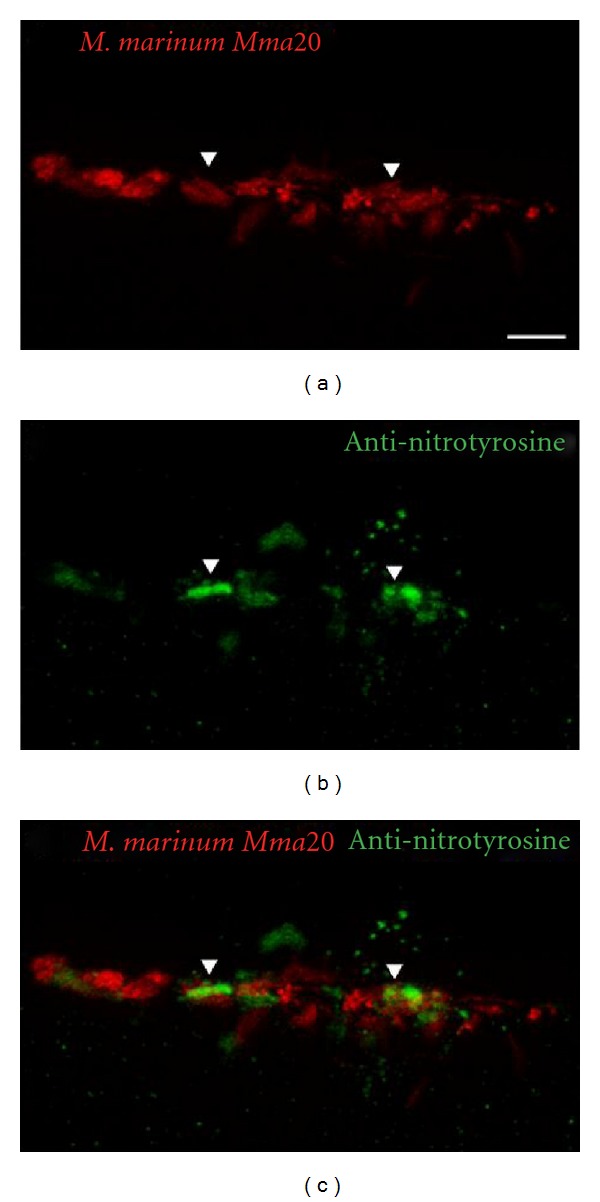
*In situ* detection of reactive nitrogen species. Wild-type zebrafish embryos (Albino; 28 hpf) were injected into the caudal vein with 200 colony-forming units (CFU) of *M*. *marinum Mma20 *expressing a pMST3::mCherry vector. Confocal images were taken of a tail region of the developing larva at 3 days after infection (3 dpi), a point at which the *M*. *marinum* infection (a) has been established. Embryos were fixed in 4% paraformaldehyde at 3 dpi, and immunohistochemistry was performed, using an antinitrotyrosine antibody that detects tissue nitration (b) [21]. Colocalization (c) between bacteria and extensive tissue nitration can be observed at this time point. Scale bar: 10 *μ*m.

The zebrafish homolog of MPO, officially named MPX, is specifically expressed in neutrophils during embryonic development. Transgenic reporter lines driven by the *mpx* promoter have made the zebrafish a highly suitable model organism to study neutrophilic inflammation [8, 171]. In fact, using one of these lines, it was demonstrated for the first time that H_2_O_2_ produced in the context of wounding not only functions as an antiseptic compound, but also forms a gradient that is required for rapid attraction of leukocytes [172]. However, this H_2_O_2_ gradient is only generated at wounds and does not occur at infected tissues [173]. The formation of this H_2_O_2_ gradient was shown to be dependent on the oxidase activity of Duox. The Src family kinase Lyn has been identified as the redox sensor that mediates neutrophil migration towards the wound [174]. The innate immune function of Duox and the importance of ROS in zebrafish were further established by studies showing that knockdown of Duox impaired the ability of zebrafish larvae to control enteric *Salmonella* infections [175]. It has also been shown that zebrafish Phox is important in controlling the *in vivo *growth of the pathogenic fungus *Candida albicans* [176]. A 5,5-dimethyl-l-pyrroline N-oxide- (DMPO-) based immunospin trap technique has been adopted for in situ detection of ROS production in zebrafish embryos [177]. DMPO is a chemical substrate that binds to reactive oxygen, which can later be detected with an anti-DMPO antibody. This protocol detects the build-up of the conjugated product, thereby showing a cumulative ROS production. Furthermore, a respiratory burst assay has been developed for zebrafish embryos, which was used to demonstrate that macrophages and neutrophils are the ROS-producing cells in zebrafish [178]. A similar method is available to image the production of NO in zebrafish embryos, using a diaminofluorescein probe that only becomes fluorescent in the presence of NO [179]. As mentioned before, nitration of tyrosine residues is a hallmark of NO production. Forlenza et al. (2008) used an antinitrotyrosine antibody on common carp tissue to visualize the tissue nitration that occurs at sites of *Trypanoplasma  borreli* infection [21]. We used the same antibody for immunohistochemistry on zebrafish embryos to visualize the production of RNS in response to *M*. *marinum* infection (Figure 3). This technique also visualizes the nitrosative stress that the host tissue suffers upon release of RNS. The resolution of inflammation that should prevent tissue damage following such stresses has also been studied in zebrafish. This has led to new insights on the mechanisms underlying resolution, including apoptosis and retrograde chemotaxis of neutrophils, with the oxygen-sensing transcription factor hypoxia-inducible factor-1*α* (Hif-1*α*) playing a role in the control of these mechanisms [171, 180].

## 4. Gene Expression Programs Reflecting Innate Immune Responses

### 4.1. Genome-Wide Expression Profiling

The availability of the zebrafish genome sequence facilitates the use of microarray and deep sequencing techniques for genome-wide expression profiling. Zebrafish embryos and larvae are useful for *in vivo* analysis of gene expression profiles upon infection, since large numbers can be pooled to level out individual variation. However, pooling should be done with caution, and it is advisable to verify conclusions by analysis at the single-embryo level [123]. A protocol has been developed for single embryo RNA isolation that gives sufficient RNA for microarray or RNA sequencing [181]. Expression profiling can be done either at whole organism level or on FACS-sorted immune cells from transgenic lines. The latter approach was used to determine the transcriptional signature of early myeloid cells [87]. Microarray analysis of zebrafish adults and embryos infected with various pathogens has provided insights into the transcriptome during infection and has provided leads for further functional studies (Table 1). The transcriptional response of both zebrafish embryos and adults showed clear conservation with host responses detected in other vertebrate models and human cells. Genes that were induced upon infection included receptors involved in pathogen recognition, signaling intermediates, their downstream transcription factors (like NF*κ*B and AP-1), and inflammatory mediators. Furthermore, these studies led to the identification of novel immune responsive genes and infection markers, for example, the DNA-damage-regulated autophagy modulator 1 gene (*dram1*), which was identified in a knockdown study of Traf6, a central intermediate in TLR and TNF receptor signaling [37].

### 4.2. Comparison of Gene Expression Profiles Induced by Different Bacterial Pathogens

**Figure 4:**
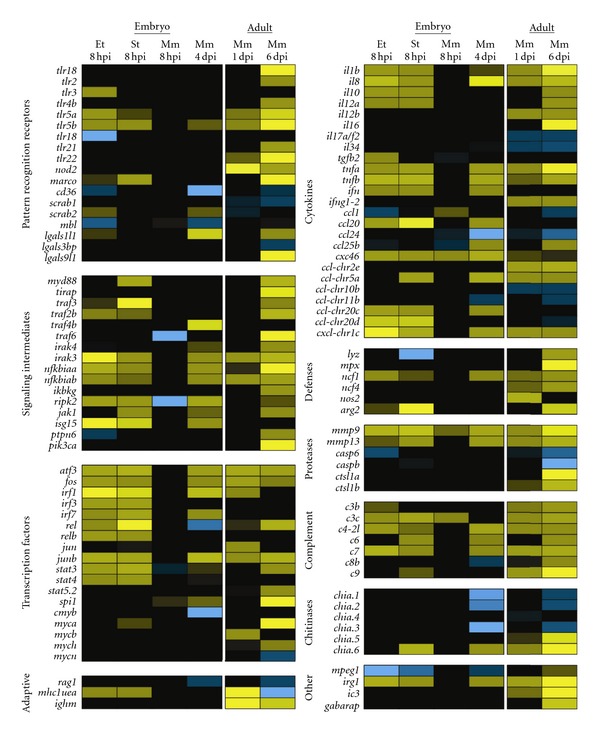
Comparison of the zebrafish innate immune response to different bacterial pathogens. Gene expression profiles of zebrafish embryos and adults infected with *E*. *tarda FL6-60 *(Et), * S*. *typhimuriumSL*1027(St), and *M*. *marinum Mma20 *(Mm) are depicted in a heat map. Embryos were infected with 200 CFU of each pathogen into the caudal vein at 28 hpf and snap frozen individually at 8 hpi for *E.  tarda* and *S*. *typhimurium*, and at 8 hpi and 4 dpi for *M*. *marinum*. Triplicate samples for each infection condition were compared with samples from control embryos (injected with PBS) using a common reference microarray design. The raw data were deposited in the Gene Expression Omnibus database under accession number GSE35474. The data derived from embryonic infections were compared with data from a study in which adult zebrafish were infected intraperitoneally with *M*. *marinum Mma20, *after which RNA samples were taken at 1 dpi and 6 dpi [22]. The dose of the *Mma20* strain used in the adult infection study was lethal within days after the final sampling point at 6 dpi. Only genes relevant to this paper were included in the heatmap. All selected genes are represented by a minimum of two probes that showed significant up or downregulation (significance cut-offs for the ratios of infected versus control groups were set at 2-fold with *P* < 10^−5^). Upregulation is indicated by increasingly bright shades of yellow, and downregulation is indicated by increasingly bright shades of blue. It should be noted that the genes listed in this figure are named according to sequence homology with mammalian counterparts and in most cases have not yet been confirmed functionally.

To illustrate the similarities and differences in the innate immune response against different bacterial pathogens, Figure 4 shows a comparison of the gene expression profiles of zebrafish infected with *Edwardsiella tarda*, *S*. *typhimurium*, and *M*. *marinum*. *E*. *tarda* is a Gram-negative, naturally occurring fish pathogen that belongs to the Enterobacteriaceae family. Inside its host, *E*. *tarda* is able to resist complement activity and can survive inside macrophages [184]. It causes a progressive disease when injected into the caudal vein of 28 hours after fertilization (hpf) embryos, leading to mortality within 2 days after infection (dpi) [123]. *S. typhimurium *(short for *S*. *enterica* serovar Typhimurium), also belonging to the Gram-negative Enterobacteriaceae family, causes salmonellosis in a broad range of hosts. *S*. *typhimurium* is a facultative intracellular species that can survive within phagocytic and nonphagocytic cells. Following internalization, it survives and replicates in a modified phagosome, known as the *Salmonella*-containing vacuole. Like *E*. *tarda*, injection of *S*. *typhimurium* into the caudal vein at 28 hpf leads to a progressive disease which leads to mortality of the embryo during the first 30 hours after infection (hpi) [13, 185]. In contrast, *M*. *marinum* injection at the same stage leads to a chronic infection that persists during larval development. *M*. *marinum* is a natural pathogen of teleost fish and a close relative of *M*. *tuberculosis*, the causative agent of tuberculosis in humans. *Mycobacteria* have a thick, waxy, acid-fast staining cell wall containing characteristic lipids that are important for virulence. Both *M*. *marinum* and *M*. *tuberculosis* have the ability to replicate inside macrophages, eventually causing them to undergo apoptosis. Dependent on secreted virulence factors that are conserved between *M*. *marinum* and *M*. *tuberculosis*, other macrophages are attracted to the initial infection site. These become infected by phagocytosing the apoptotic remains, which ultimately leads to the formation of a granuloma [186]. Using the zebrafish embryo model, Ramakrishan et al. have provided new insights demonstrating the importance of the innate immune system to control *M*. *marinum* infection during early stages of pathogenesis [1, 2, 124, 187, 188]. 

Complementary to previously reported transcriptome data (Table 1), here we present new data comparing the gene expression profiles induced by *E*. *tarda*, *S*. *typhimurium, *and *M*. *marinum* under similar conditions (Figure 4). We injected 200 colony-forming units (CFUs) of each pathogen into the caudal vein of 28 hpf zebrafish embryos and analyzed the response at 8 hpi. Since *M*. *marinum* develops a chronic infection, we also sampled at 4 dpi, a time point at which granulomas are present. Finally, we compared the transcriptome profile of the embryonic samples with data from a previous study, in which adult zebrafish were infected with the same strain of *M*. *marinum* [22]. 

The two progressive Gram-negative pathogens, *E*. *tarda* and *S*. *typhimurium*, induced a strong early immune response at 8 hpi, while the chronic *M*. *marinum* infection hardly induced any response at this time point. At 4 dpi, the transcriptome profile of *M*. *marinum*-infected embryos did show an immune response, although it was still weaker than the response to *E*. *tarda* or *S*. *typhimurium* infection at 8 hpi. In adults, the immune response to *M*. *marinum* infection has been shown to develop in a similar manner, with hardly any induction of proinflammatory genes at 1 dpi and a stronger response at 6 dpi, when the fish began to show symptoms of disease [22]. Infections with *E*. *tarda* and *S*. *typhimurium* resulted in a remarkably similar transcriptome. Nevertheless, subtle differences were observed, like the upregulation of Tlr3 that was specific to *E*. *tarda* infection in this data set, and the variation in the panel of cytokines expressed upon these infections. 

Interestingly, various PRRs, for example, Tlr5a and 5b, showed increased expression upon infection, most likely indicating an elevated state of awareness needed to identify the invading pathogens. In contrast, the fish-specific Tlr18, the scavenger receptors CD36, scarb1, and scarb2, and the C-type lectin Mbl were downregulated in some conditions. In many cases, signaling intermediates downstream of PRRs were upregulated, relaying and possibly amplifying the activating signals they receive from their respective receptors. A wide range of transcription factors with well-established functions in immunity (e.g., Atf3, Jun and Fos, Rel, and the IRF and Stat family members) were significantly upregulated under all conditions tested, except for the 8 hpi time point of *M*. *marinum* infection, whereas we observed upregulation of transcription factors of the oncogenic Myc family mainly in adult fish. The hematopoietic transcription factor Spi1 (Pu.1) was upregulated in *M*. *marinum* infection of embryos and adults. Genes for the key pro-inflammatory cytokines, like TNF*α* (two genes in zebrafish: *tnfa* and *tnfb*), IL1*β*, and IL8, and for the anti-inflammatory cytokine IL10 were induced by infection with any of the three pathogens. Other cytokines appeared to be more specific for certain pathogens or might not be expressed at the specific time point of infection that we sampled. 

We also observed increased expression of genes involved in effector mechanisms. However, upregulation of the genes encoding lysozyme, myeloperoxidase, and iNos was detectable only in adult zebrafish infected with *M*. *marinum*. Infection with any of the three pathogens led to increased gene expression of *ncf1*, a subunit of the neutrophil NADPH oxidase complex. Proteases are an important part of the innate immune response, functioning in reorganizing the extracellular matrix to allow leukocyte migration, in degradation of microbes, and in processing of cytokines. In adult zebrafish infected with *M*. *marinum*, we observed upregulation of cathepsin-like 1a and 1b (*ctsl1a* and *ctsl1b*), members of lysosomal cathepsin family that aids in the destruction of microbes. Expression levels of *casp6* and *caspb, *members of the cysteine-aspartic acid protease (caspase) family involved in apoptosis, were downregulated at different stages of infection in adults and embryos. The matrix metalloproteinase (mmp) genes 9 and *mmp13* proved to be excellent markers for infection, since their gene expression was induced by *E*. *tarda*, *S*. *typhimurium* and *M*. *marinum*.

Our data further suggest that complement activation plays an important role during the early innate immune response, since a large number of complement factor genes show increased expression upon infection. Upregulated expression of the autophagy marker genes *lc3* and *gabarap* in adults infected with *M*. *marinum* hints towards a role for autophagy in the control of this infection. Intriguingly, a macrophage-expressed gene with unknown function in immunity, *mpeg1 *[87], is downregulated during the embryonic immune response against all three pathogens. The mouse homolog of this gene encodes a perforin-like protein that is expressed in mature macrophages and prion-infected brain cells [189]. We have also observed specific upregulation of genes with as of yet unknown function in immunity, like immunoresponsive gene 1 (*irg1*). This gene is highly conserved in vertebrates and has high homology to bacterial methylcitrate dehydrogenase [190]. We also included some genes involved in adaptive immunity in our comparison, the lymphocyte marker *rag1*, the immunoglobulin heavy chain gene *ighm,* and the antigen-presenting major histocompatibility complex class I UEA gene (*mhc1uea*). Even though no cells of the adaptive immune system are present yet, embryos infected with *E*. *tarda* or *S*. *typhimurium* increase the expression of the MHC I gene. Finally, upon infection with *S*. *typhimurium* and *M*. *marimum*, we observe up and downregulation of chitinases, a family of genes which has been attributed a role during the host-microbial interactions involved in the development of acute and chronic inflammatory conditions [191].

## 5. Discussion

Zebrafish infectious disease models have started to make an important contribution to the understanding of host-pathogen interaction mechanisms. A good example is the discovery of the mechanism whereby a mycobacterial virulence factor (ESAT6) induces *mmp9* expression in host epithelial cells neighboring infected macrophages, which enhances macrophage recruitment and formation of granuloma-like aggregates that provide a replication niche for mycobacteria [2]. The combination of genetics and *in vivo* imaging in zebrafish embryos is unparalleled in other vertebrate models. Furthermore, zebrafish embryos provide an ideal model for high-throughput *in vivo* screening of antimicrobial drug candidates or novel vaccine candidates [192, 193]. Knowledge of the zebrafish immune system is also important in high-throughput screening for cancer in zebrafish embryos [194]. However, many aspects of zebrafish immunity still require further characterization and validation. 

Currently available transgenic lines clearly distinguish macrophages (marked by *csf1r*/*fms* and *mpeg1*) from neutrophils (marked by *mpx* and *lyz*) in embryos and larvae, but there is insufficient knowledge of surface markers to identify different macrophage and neutrophil subpopulations. Similar to mammals, there is evidence of the existence of subpopulations of classically activated macrophages (M1: high producers of proinflammatory mediators, ROS, and NO) and alternatively activated macrophages (M2: high producers of anti-inflammatory mediators) in fish [195]. The polarization of macrophages towards these subtypes plays a critical role in the pathology of both infectious diseases and cancer [196]. Furthermore, different subpopulations of mammalian neutrophils (N1 and N2) have been recently described that display pro- and antitumorigenic properties [197] and that probably will also turn out to have distinctive functions during infectious disease pathology. Tumor implants in zebrafish embryos were shown to attract a heterogeneous population of leukocytes, including cells that express arginase, a marker of alternatively activated macrophages [177]. In addition, the neutrophil markers *mpx, mych*, and *lyz* do not show complete overlap [177, 198], and markers such as *cxcr3*.2 and *ptpn6*, which are macrophage specific in one-day-old embryos, also label a subset of neutrophils at later stages [87]. Future development of transgenic lines that can distinguish these multiple myeloid subsets would further strengthen the use of zebrafish models for innate immunity and infectious disease studies.

As detailed in this paper, counterparts of the major vertebrate PRRs and downstream signaling components have been identified in zebrafish, but relatively few have thus far been functionally studied in infectious disease models. Recently, new PRRs have been described in mammals, like the INF-inducible dsRNA-activated protein kinase R (PKR) [199], the cytosolic DNA sensor DNA-dependent activator of IFN-regulatory factors (DAI) [200], and a cytosolic DNA receptor named AIM2 (absent in melanoma 2) [201]. Thus far, only the zebrafish homolog for PKR has been identified. Furthermore, autophagic adaptors known as sequestosome 1/p62-like receptors (SLRs), conserved between zebrafish and human, have recently been suggested as a new category of PRRs, since they have the ability to recognize and capture targets for immune-related autophagy [202].

Various datasets derived from transcriptome analyses have shown the specificity of immune responses to different pathogens. In future studies, the analysis of these responses can be refined by FACS sorting of immune cell populations from infected embryos, using labeled pathogens in combination with transgenic lines for different immune cell types. For example, it now comes within reach to aim at dissecting the differences in gene expression between *M*. *marinum*-infected macrophages inside a granuloma and recently attracted uninfected macrophages. In addition, simultaneous profiling of pathogen and host genes will be a challenging approach to help unravel the complex mechanisms underlying host-pathogen interactions. Transcriptome analysis only reveals altered RNA levels upon infection, and therefore, the application of proteomic and epigenetic analyses are needed to study the regulation of immune responses on different levels. Transcriptome studies have revealed infection responsiveness of many genes that have not yet been well studied (for example, *dram1*, *mpeg1*, *irg1*, and *irg1l*, mentioned above) and an emerging immune function for several chitinase-like proteins during infection [13, 37, 123]. Many zebrafish infection models have been described here and in other recent papers [4, 203, 204] that can be used to investigate the functions of these genes in different pathogenic interactions, either using morpholino knockdown in embryos or using stable knockout lines which nowadays can be identified very efficiently by high-throughput resequencing of mutant libraries or by targeted knock-down approaches using technologies such as zinc finger nucleases (ZFNs) or transcription activator-like effector nucleases (TALENs) [205]. 

## Figures and Tables

**Table 1:** Transcriptome profiling studies on infection models in adult and embryonic zebrafish.

Bacterial species	Strain	Infection model	Reference
*Mycobacterium marinum*	M; E11	Adult (IP)	Meijer et al.^∗^[182]
*Mycobacterium marinum*	*Mma*20; E11	28hpf (CV); Adult (IP)	Van der Sar et al. [22]
*Mycobacterium marinum*	M; E11	Adult (IP)	Hegedus et al.^∗^ [107]
*Salmonella enterica serovar Typhimurium * (*Salmonella typhimurium*)	SL1027; LPS derivative SF1592 (*Ra*),	28hpf (CV)	Stockhammer et al.^∗∗^ [13]
*Streptococcus suis*	HA9801	Adult (IP)	Wu et al. [183]
*Salmonella enterica serovar Typhimurium * (*Salmonella typhimurium*)	SL1027; LPS derivative SF1592 (*Ra*),	28hpf (CV)	Ordas et al.^∗∗^ [38]
*Edwardsiella tarda*	FL6-60	28hpf (CV)	Van Soest et al. [123]
*Citrobacter freundii*	Not specified	Adult (IM)	Lu et al. [153]

^∗^ and ^∗∗^: these studies used the same samples but applied microarray analysis and deep sequencing, respectively.

(IP): intraperitoneal; (CV): caudal vein; (IM): immersion.
